# Serum fibroblast growth factor-2 levels complement vital biomarkers for diagnosing heart failure

**DOI:** 10.1186/s12872-024-03768-4

**Published:** 2024-02-15

**Authors:** Z. L. Yu, Z. H. Cai, J. T. Zheng, H. Y. Jiang, Y. Q. Zhou, N. K. Wong, H. B. Fu, X. B. Hong

**Affiliations:** 1 Department of Pharmacy, The Second Affiliated Hospital, Shantou University Medical College, Shantou, China; 2 Department of Pharmacy, Cancer Hospital of Shantou University Medical College, Shantou, China; 3 Department of Pharmacology, Shantou University Medical College, Shantou, China

**Keywords:** Biomarker, Left ventricular ejection fraction, Fibroblast growth factor-2, Heart failure, Cardiac hypertrophy

## Abstract

**Background:**

Early diagnosis of atrial fibrillation is important as it is crucial for improving patient outcomes. Fibroblast growth factor-2 (FGF2) may serve as a diagnostic biomarker for heart failure due to its ability to promote cardiac fibrosis and hypertrophy; however, the relationship between FGF2 concentration and heart failure is unclear. Therefore, this study aimed to explore whether FGF2 could aid in distinguishing patients with heart failure from healthy controls and those with dyspnea without heart failure. Additionally, to evaluate the possible correlation between serum FGF2 levels and its diagnostic parameters in patients with heart failure.

**Methods:**

Plasma FGF2 concentration was measured in 114 patients with a complaint of dyspnea (enrolled in the study between January 2022 and August 2022). Based on heart failure diagnosis, the patients were assigned to three groups, as follows: heart failure (*n* = 80), non-heart-failure dyspnea (*n* = 34), and healthy controls (*n* = 36), following physical examination. Possible correlations between serum FGF2 levels and other prognostic parameters in patients with heart failure were analyzed.

**Results:**

Serum FGF2 levels were higher in patients with heart failure (125.60 [88.95, 183.40] pg/mL) than in those with non-heart-failure dyspnea (65.30 [28.85, 78.95] pg/mL) and healthy controls (78.90 [60.80, 87.20] pg/mL) (*p* < 0.001). Receiver operating characteristic curve analysis identified FGF2 concentration as a significant predictor in heart failure diagnosis, with an area under the curve of 0.8693 (*p* < 0.0001). Importantly, in the heart failure group, serum FGF2 concentrations correlated with key prognostic parameters for heart failure, such as reduced left ventricular ejection fraction and elevated serum levels of *N*-terminal pro-B-type natriuretic peptide.

**Conclusions:**

Elevated serum FGF2 level is strongly associated with an increased risk of heart failure and could serve as a useful biomarker to complement vital diagnostic parameters for heart failure.

**Supplementary Information:**

The online version contains supplementary material available at 10.1186/s12872-024-03768-4.

## Background

Heart failure (HF) is a complex, life-threatening clinical syndrome with high prevalence, incidence, mortality, and health care costs and a profound socioeconomic burden worldwide [1]. It is currently defined as “a clinical syndrome with symptoms and/or signs caused by a structural and/or functional cardiac abnormality and corroborated by elevated natriuretic peptide levels and/or objective evidence of pulmonary or systemic congestion” according to the European Society of Cardiology guidelines [2]. HF prevalence in developed countries is 1.5–2.0%, and it accounts for substantial morbidity and mortality worldwide [2, 3]. Therefore, prediction, early diagnosis, and treatment guidance are crucial.

Myocardial fibrosis is closely related to the occurrence and development of HF [2]. Specific biomolecules involved in pathophysiological processes, such as myocardial fibrosis and remodeling, may serve as HF biomarkers [4, 5]. Fibroblast growth factor-2 (FGF2) is a broad-spectrum, potent, mitogenic and angiogenic factor [6] essential in heart development, homeostasis, disease, and repair [7]. FGF2 can promote cardiac hypertrophy and fibrosis [8–11] through its high-molecular-weight FGF2 isoform (hi-FGF2) [12–14]. Our previous study reveals a novel mechanism, by which cells overexpressing hi-FGF2 can induce mitochondria-associated apoptosis which is closely associated with HF [15].The level of hi-FGF2 can serve as a prognostic biomarker to predict the occurrence of HF in patients with atrial fibrillation (AF) [15, 16]. However, the relationship between FGF2 concentration and HF is unclear.

We hypothesized that the FGF2 level may be an HF biomarker due to its ability to promote cardiac fibrosis and hypertrophy. Hence, we aimed to evaluate and compare serum FGF2 levels in patients with HF, those having dyspnea without HF, and healthy controls. To the best of our knowledge, this is the first study to compare FGF2 levels between these groups of individuals. Furthermore, we calculated the area under the receiver operating characteristic curve (AUC) to shed light on the utility of FGF2 levels in HF diagnosis. Similarly, we explored the correlation of FGF2 levels with known prognostic clinical markers of HF, such as N-terminal pro-B-type natriuretic peptide (NT-proBNP) concentration and cardiac function parameters (left ventricular ejection fraction [LVEF]), to determine the ability of FGF2 expression level to indicate HF severity.

## Methods

### Study population

We enrolled 36 healthy volunteers and 114 patients admitted to the hospital for dyspnea between January 2022 and August 2022. Among the patients with dyspnea, 80 and 34 were determined to have HF and normal cardiac function, respectively. General demographic data were collected. This study was approved by the Ethics Committee of the Second Affiliated Hospital of Shantou University Medical College, Shantou, China (NO. 2021–13), and conducted in accordance with the principles outlined in the Declaration of Helsinki. Plasma was obtained from patients. Written informed consent was obtained from all patients at the study entry.

Inclusion criteria for the HF group were consistent with those of the Framingham diagnostic criteria. If HF was excluded through clinical diagnosis and dyspnea symptoms were observed, a patient was assigned to the non-HF dyspnea group. Unknown coronary, valvular, or myocardial diseases did not affect the enrolment of healthy controls. Exclusion criteria for all groups included the presence of rheumatic heart disease, severe liver and kidney diseases, immune dysfunction, pregnancy, malignancy, or acute myocardial infarction (MI).

Demographic, clinical, and baseline biochemical data were collected for all participants. In addition, echocardiographic parameters in the HF and dyspneic non-HF groups were collected to identify cardiac hypertrophy.

### Measurement of serum FGF2 concentration

Baseline peripheral venous blood was collected from all participants in a vacuum tube containing potassium ethylene diamine tetraacetic acid, naturally solidified at 37°C for 30 min, and centrifuged at 1000 g for 15 min. Separated serum was placed in a centrifuge tube and stored at − 80 °C. FGF2 content was measured using an enzyme-linked immunosorbent assay kit (Proteintech Group, Rosemont, IL, USA; stock number EK0342) and Tecan.

### Statistical analysis

All statistical analyses were performed using SPSS 26.0 software (version 22.0; IBM Corporation, Armonk, NY, USA) and GraphPad Prism 8 software (GraphPad Software, Inc., Boston, MA, USA). Continuous variables with normal distribution are expressed as means and standard deviations (x ± s), and comparisons between multiple groups were performed using one-way analysis of variance and pairwise comparisons using the Student–Newman–Keuls method. Non-normally distributed variables are expressed as medians and interquartile ranges. The rank sum test was used for comparisons between multiple groups. Categorical clinical variables are presented as counts (percentages) and were compared using the chi-square test.

The capacity of FGF2 level to discriminate between HF, dyspnea without HF, and healthy controls was characterized using a receiver operating characteristic curve, and the AUC was calculated. Considering the relationship between serum FGF2 levels and other variables, such as LVEF and NT-proBNP concentration, was non-linear, Spearman’s correlation coefficient was used to assess these associations. Statistical significance was determined at *p* < 0.05.

## Results

### Characteristics of the study population

**Fig. 1:**
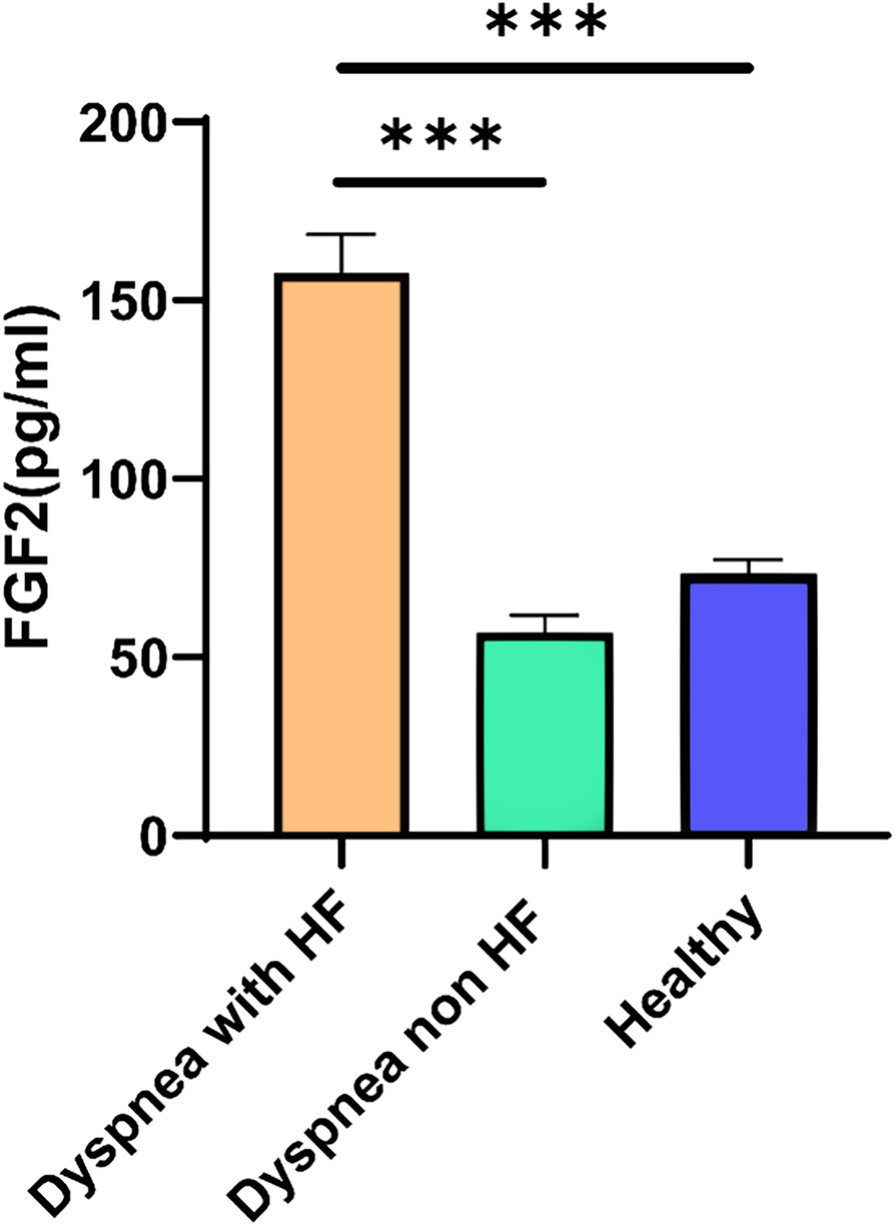
FGF2 levels in patients with HF, those having dyspnea without HF, and healthy controls. Data represent mean ± SD (*n* = 3). Statistical significance was calculated via one-way analysis of variance, * *p* < 0.05; ** *p* < 0.01; *** *p* < 0.001. Abbreviations: FGF2, fibroblast growth factor 2; HF, heart failure; SD, standard deviation

This study included 114 patients with dyspnea diagnosed with (*n* = 80) and without (*n* = 34) HF, along with 36 healthy controls. The clinical characteristics and laboratory findings of each group are shown in Table 1. No significant differences between groups were observed regarding diastolic blood pressure, hemoglobin level, platelet count, and serum sodium, chloride, and phosphorus concentration (*p* > 0.05). However, significant between-group differences were observed in smoking habits, comorbidities (including hypertension, diabetes, coronary heart disease, chronic kidney disease, atrial fibrillation, MI, and cerebral infarction), drug regimens, white blood cell and neutrophil counts, and high- and low-density lipoprotein cholesterol levels. LVEF% was significantly reduced in patients with HF than in patients with non-HF dyspnea, LVEDD, LVESD, E/E’ and LVM(g) were similarly raised in the HF group. Meanwhile, biochemical indicators, such as cystatin C, FGF2, NT-proBNP, creatinine, and high-sensitivity C-reactive protein, were significantly higher in patients with HF. FGF2 expression levels in each group are shown in Fig. 1. No significant differences were observed in FGF2 expression between patients with dyspnea without HF (65.30 [28.85, 78.95] pg/mL) and healthy controls (78.90 [60.80, 87.20] pg/mL) (*p* > 0.05). FGF2 concentration was elevated in the HF group (125.60 [88.95, 183.40] pg/mL) compared with that in the non-HF dyspnea and healthy control groups (*p* < 0.001).

**Table 1:** Baseline demographic and clinical characteristics of study participants

characteristic	Healthy Controls(n = 36)	Non-HF Cases(n = 34)	HF cases(n = 80)	*P*
Age(years)	54.80 ± 8.88	59.26 ± 11.80	60.90 ± 9.62*	< 0.001
Male,n(%)	13(36.11)	21(61.76)	55(68.75)*	< 0.001
BMI(kg/m^2^)	22.67(20.98,25.81)	24.44(22.35,25.77)	24.03(21.95,27.89)	0.512
Current smoking(%)	1(2.78)	9(26.47)	32(40.00)*	0.013
SBP(mm Hg)	128.24 ± 20.26	146.44 ± 22.72	141.14 ± 25.76	0.045
DBP(mm Hg)	80.00(70.00,90.00)	83.00(77.75,88.25)	81.50(75.25,96.25)	0.675
Medical history				
Hypertension,n(%)	0(0.00)	24(70.58)	58(72.50)*	< 0.001
DM,n(%)	0(0.00)	10(29.41)	23(28.75)	0.001
CHD,n(%)	0(0.00)	9(26.47)	44(55.00)*#	< 0.001
Chronic renal failure,n(%)	0(0.00)	1(2.94)	11(13.75)	0.017
AF,n(%)	0(0.00)	0(0.00)	24(30.00)*#	< 0.001
Old myocardial infarction,n(%)	0(0.00)	0(0.00)	14(17.50)*#	0.001
Cerebral stroke,n(%)	1(2.78)	7(20.59)	17(21.25)*	0.037
NYHA grade				
Class I	–	–	11(17.2)	
Class II	–	–	22(34.4)	
Class III	–	–	21(32.8)	
Class IV	–	–	8(12.5)	
WBC(× 10^9^/L)	5.75(4.92,7.02)	6.90(5.90,7.90)	7.85(6.00,9.70)*	< 0.001
NEUT(×10^9^/L)	3.47(2.70,4.10)	4.36(3.35,5.20)	5.14(3.47,7.21)*	< 0.001
RBC(× 10^12^/L)	4.69 ± 0.52	4.60 ± 0.56	4.40 ± 0.66*	0.048
Hemoglobin,(g/L)	133.50(127.50,138.50)	138.00(129,144)	130.50(120.75,142.5)	0.147
PLT(×10^9^/L)	230.50(199.25,270.50)	223.00(180,278.5)	207.00(169.75,250.25)	0.092
Cystatin C(mg/L)	0.83(0.78,0.89)	0.87(0.80,1.12)	1.20(1.01,1.43)*#	< 0.001
NT-proBNP(pg/mL)	100.00(100,100)	100.00(100,218.25)	3453.00(676.50,8293.75)*#	< 0.001
FGF2(pg/mL)	78.90(60.80,87.20)	65.30(28.85,78.95)	125.60(88.95,183.4) *#	< 0.001
K^+^(mmol/L)	4.02(3.83,4.12)	3.79(3.64,3.90)	3.81(3.45,4.04)	0.039
Na^+^ (mmol/L)	139.70(139.2140.60)	140.20(138.77,141.45)	140.30(138.1142.2)	0.734
Cl^−^(mmol/L)	103.40(102.00,104.45)	104.05(101.57,105.02)	103.20(100.4106.1)	0.719
Ca^2+^ (mmol/L)	2.41 ± 0.08	2.35 ± 0.10	2.27 ± 0.12*#	< 0.001
P^−^(mmol/L)	1.18 ± 0.14	1.12 ± 0.19	1.12 ± 0.20	0.768
Creatinine (μmol/L)	80.70(68.45,92.25)	81.05(70.75,94.77)	100.00(88.5116.4)*#	< 0.001
BUN(mmol/L)	5.56(4.68,6.10)	5.00(3.98,5.82)	6.46(4.96,7.90)*#	< 0.001
TC(mmol/L)	5.30(4.49,5.89)	5.28(4.03,5.97)	4.31(3.34,5.29)*#	0.001
TG(mmol/L)	0.98(0.78,1.31)	1.35(0.82,2.27)	1.04(0.80,1.64)	0.042
HDL-C(μmol/L)	1.44(1.15,1.78)	1.09(0.95,1.37)	1.03(0.88,1.34)*	< 0.001
LDL-C(μmol/L)	3.42 ± 0.77	3.39 ± 0.96	2.90 ± 1.03*#	0.012
hs-CRP(mg/L)	1.19(0.47,2.55)	2.02(0.62,6.08)	4.30(1.44,21.3)*#	0.006
Fg(g/L)	4.06(3.68,4.43)	3.98(3.71,4.24)	4.07(3.57,4.46)	0.980
Myocardial hypertrophy,n(%)	0(0.00)	1(3.20)	23(29.10)*#	< 0.001
EF				0.014
> 45%	6(100.00)	31(100.00)	64(80.00)#	
< 45%	0(0.00)	0(0.00)	16(20.00)#	
Echocardiographic parameters				
LVEF (%)		64.00(62.00,67.00)	56.00(48.00,62.00)#	< 0.001
LVEDD(mm)	–	47.09 ± 3.95	49.00(46.00,53.00)#	0.002
LVESD(mm)	–	30.59 ± 3.39	33.00(31.00,37.00)#	< 0.001
LVFS(%)	–	34.59 ± 3.26	31.0(28.00,34.00)#	< 0.001
E/A	–	0.70(0.70,0.83)	0.7(0.60,1.10)	0.793
DT(ms)	–	204.20 ± 35.96	209.13 ± 41.61	0.683
E/E’	–	9.46 ± 2.56	12.55 ± 3.73#	< 0.001
IVST(mm)	–	9.95 ± 1.41	10.00(9.00,12.00)	0.366
LVPWT(mm)	–	9.00 ± 1.04	9.00(8.0,10.00)	0.32
LVM(g)	–	156.53 ± 39.38	175.02(147.78,232.89)#	0.001
RWT	–	0.38 ± 0.04	0.38 ± 0.09	0.557
ACEI/ARB,n(%)	0(0.00)	22(64.71)	60(75.00)*	< 0.001
β-blockers,n(%)	0(0.00)	7(20.59)	46(57.50)*#	< 0.001
Diuretics,n(%)	0(0.00)	6(17.65)	52(65.00)*#	< 0.001
Statin,n(%)	0(0.00)	20(58.82)	60(75.00)*	< 0.001
Etiology				
Ischemic etiology, n(%)	–	–	44(55.00)	
Non-ischemic etiology,n(%)	–	–	36(45.00)	

*BMI* body mass index, *SBP* systolic blood pressure, *DBP* diastolic blood pressure, *DM* diabetes mellitus, *CHD* coronary atherosclerotic heart disease, *AF* atrial fibrillation, *NYHA* New York Heart Association, *WBC* white blood cell count, *NEUT* neutrophil count, *RBC* red blood cell count, *PLT* platelet, *BUN* blood urea nitrogen, *TC* total cholesterol, *TG* triglyceride, *HDL-C* high density lipoprotein-cholesterol, *LDL-C* low density lipoprotein-cholesterol, *hs-CRP* high-sensitivity C-reaction protein, *Fg* fibrinogen, *EF* left ventricular ejection fraction, A, peak late wave diastolic filling velocity; DT, deceleration time; E, peak early wave diastolic filling velocity; E’, peak early diastolic mitral annulus velocity; IVST,interventricular septal thickness; LVEDD, left ventricular end-diastolic diameter; LVEF,left ventricular ejection fractions; LVESD,left ventricular end-systolic diameter; LVFS,left ventricular fractional shortening; LVM,left ventricular mass; LVPWT,left ventricular posterior wall thickness; RWT,relative wall thicken; *ACEI/ARB* angiotensin converting enzyme inhibitors/angiotensin receptor blocker. * *P* < 0.05 compared to healthy controls; # *P* < 0.05 compared to dyspneic non-HF cases

### Diagnostic accuracy of FGF2 concentration

**Fig. 2:**
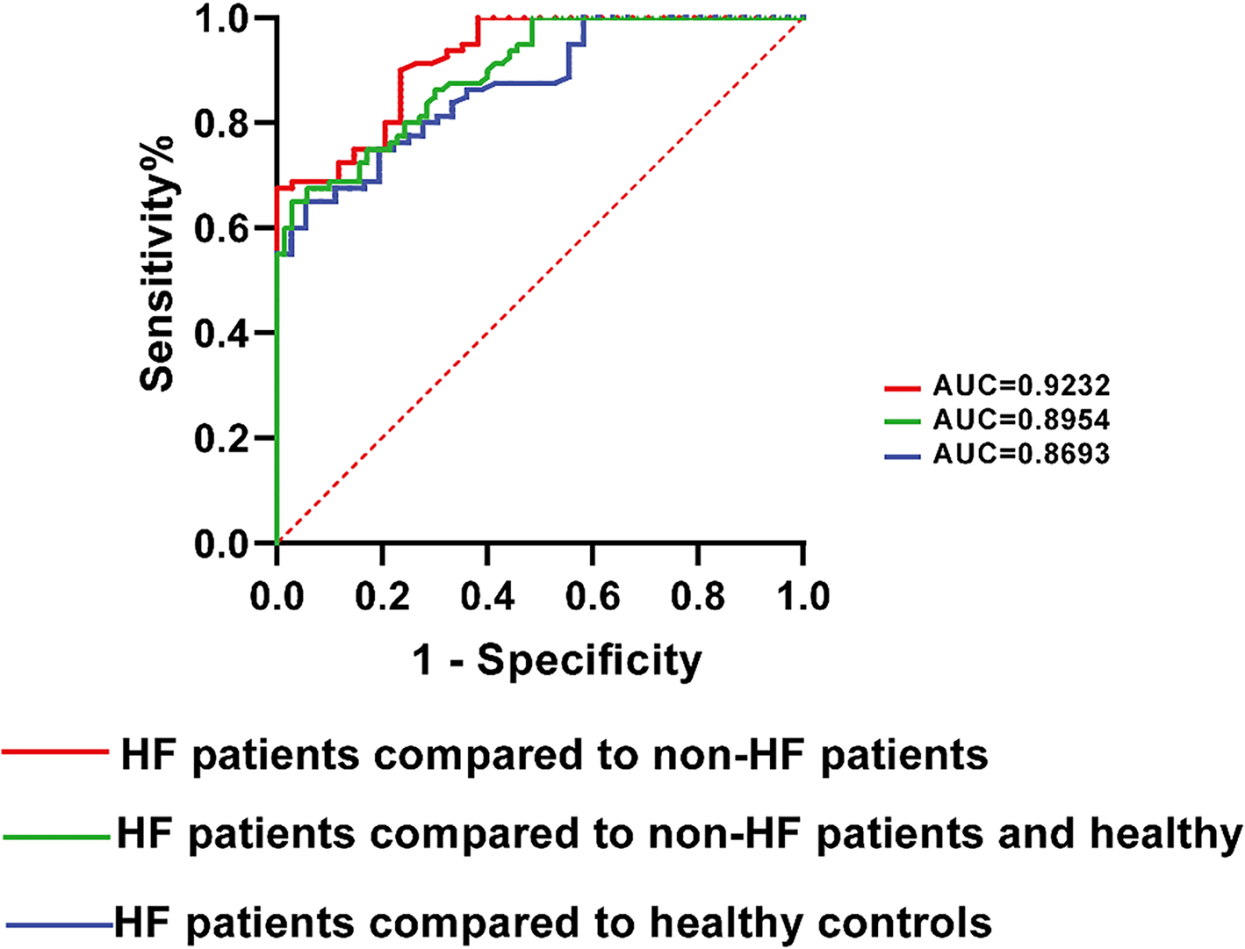
ROC curve analysis of FGF2 concentration for distinguishing HF dyspnea, non-HF dyspnea, and healthy controls. Abbreviations: ROC, receiver operating characteristic; FGF2, fibroblast growth factor 2; HF, heart failure

The AUC value of FGF2 concentration for distinguishing patients with HF and healthy controls was 0.8693 (95% confidence interval [CI]: 0.8064–0.9322), whereas the value distinguishing patients with HF, non-HF dyspnea, and healthy controls was 0.8954 (95% CI: 0.8486–0.9420) (Fig. 2). Similarly, the AUC value distinguishing HF from non-HF dyspnea was high, suggesting the robustness of the diagnostic power of FGF2 concentration (AUC, 0.9232; 95% CI: 0.8749–0.9715).

### Correlation of FGF2 with other HF parameters

**Fig. 3:**
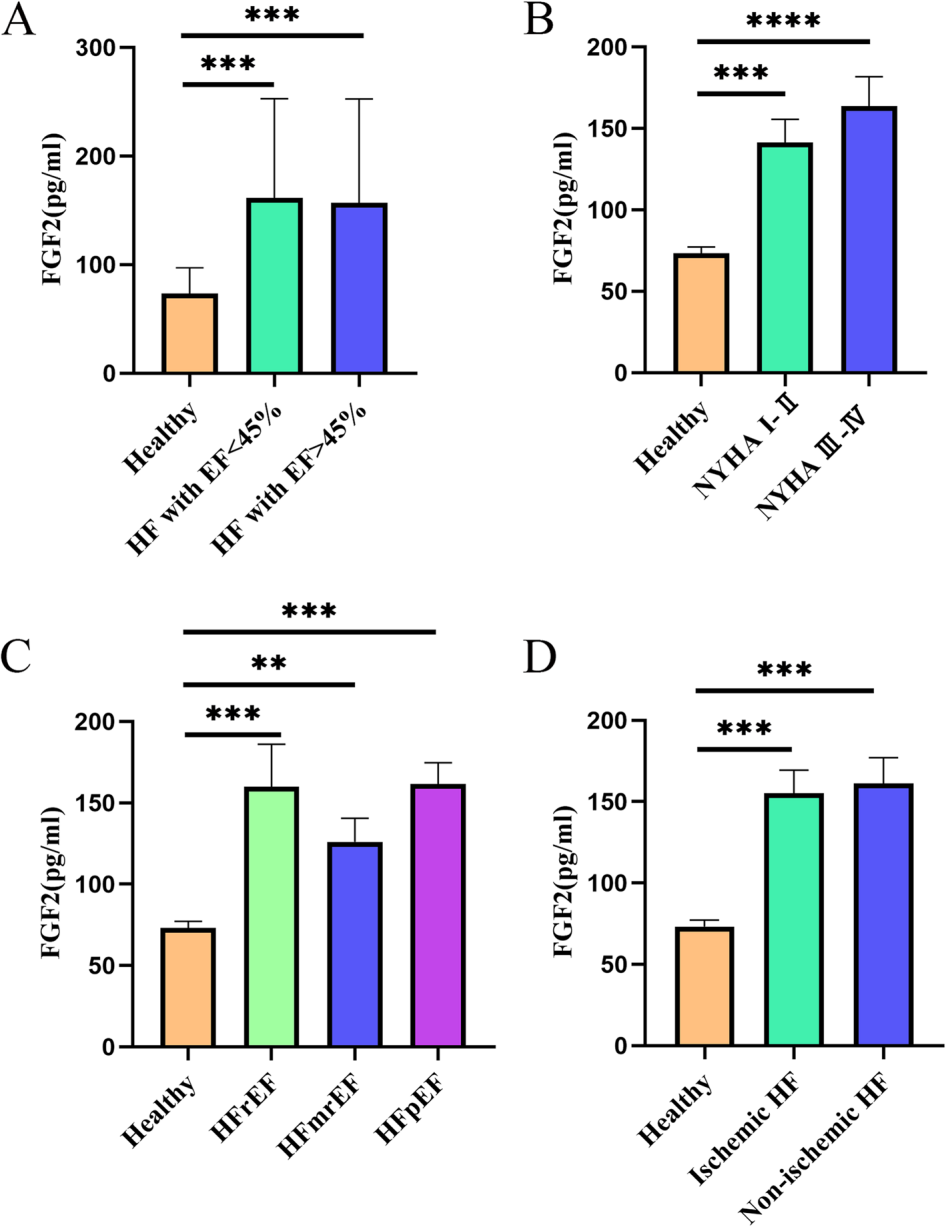
Correlation of FGF2 with other HF parameters. (**a**) Circulating levels of FGF2 in healthy controls (*n* = 36) and HF cases with ejection fraction (EF) < 45% (*n* = 16) and EF > 45% (*n* = 64). (**b**) Expression levels of FGF2 according to New York Heart Association cardiac function classification. (**c**) Expression levels of FGF2 in different HF types. (**d**) Expression levels of FGF2 in groups with different HF etiologies. ** p < 0.01; *** p < 0.001. Abbreviations: FGF2, fibroblast growth factor 2; HF, heart failure; HFrEF, heart failure with reduced ejection fraction; HFmrEF, heart failure with mildly reduced ejection fraction; HFpEF, heart failure with preserved ejection fraction

To investigate whether FGF2 levels are associated with disease severity, HF type, or HF etiology, we classified patients with HF according to LVEF, New York Heart Association (NYHA) grade, HF type, and underlying etiology. The results are illustrated in Fig. 3. No differences were observed in FGF2 levels between the LVEF > 45% and LVEF < 45% groups (*p* > 0.05) nor between the different NYHA groups. Although the level of circulating FGF2 increased as the NYHA grades increased (Ι–III), no significant differences were observed between the groups (*p* > 0.05). FGF2 levels are not significantly elevated in the HF(+)AF(+) cohort compared to the HF(+)AF (−)group(*p* > 0.05) (Fig. S1). Additionally, patients with different HF types (reduced ejection fraction, mildly reduced ejection fraction, and preserved ejection fraction) and etiologies did not differ significantly (*p* > 0.05). These results suggest that FGF2 concentration is limited in distinguishing HF with different severities, types, and causes.

**Fig. 4:**
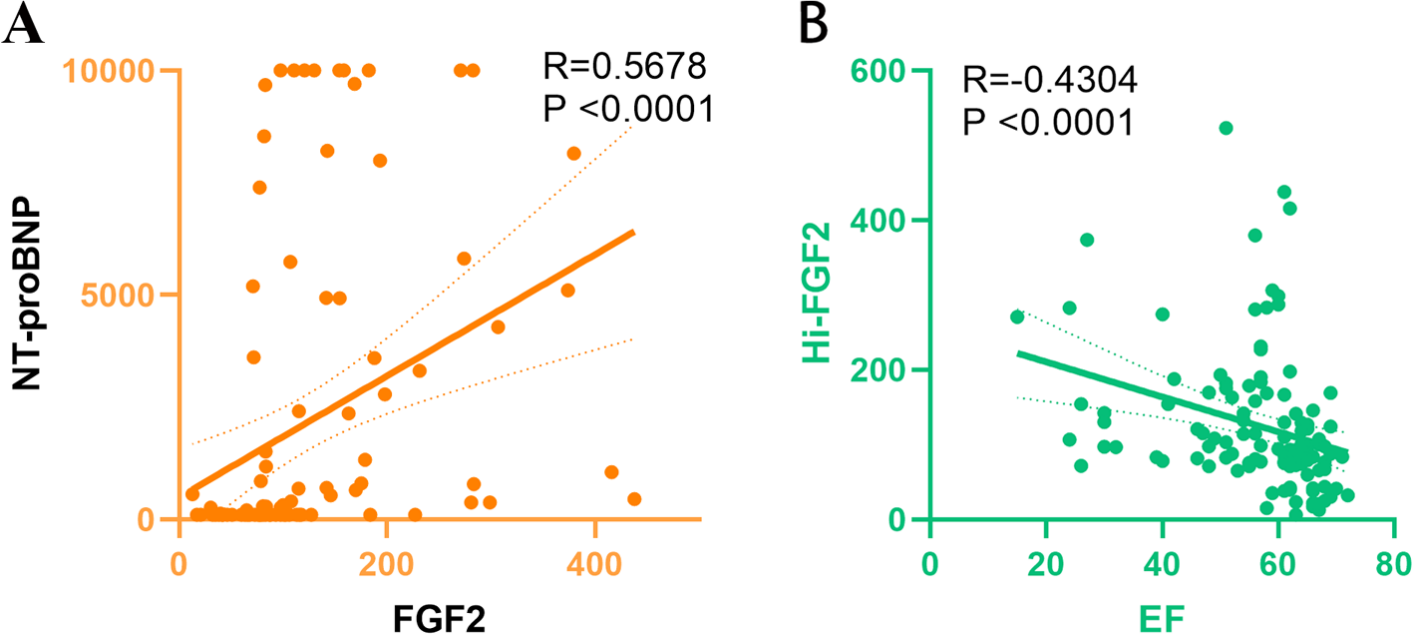
Spearman correlation of FGF2 level with N-terminal pro-B-type natriuretic peptide level (**a**) and EF (**b**). Abbreviations: FGF2, fibroblast growth factor 2; EF, ejection fraction

To further explore whether serum FGF2 concentration is associated with functional changes in the heart and HF severity, the correlations of FGF2 concentration with LVEF and NT-proBNP concentration were analyzed. Serum FGF2 concentration had a weak negative correlation with LVEF (*r*_s_ = − 0.4304, *p* < 0.0001) and a moderate positive correlation with NT-proBNP concentration (*r*_s_ = 0.5678, *p* < 0.0001) (Fig. 4).

## Discussion

This study demonstrated that elevated FGF2 concentrations were observed in patients with HF relative to patients with non-HF dyspnea and healthy controls. FGF2 level correlated with crucial prognostic parameters for HF, such as reduced LVEF and elevated levels of NT-proBNP, it could serve as a vital biomarker for HF.

Mechanisms of HF are complex and related to the structure and systolic remodeling of the atrial and ventricular myocardium. Increased oxygen demand because of various causes drives the heart to initiate an adaptive remodeling process. Cardiac remodeling includes pathological cardiomyocyte hypertrophy and fibrosis. However, persistent fibrosis progression and extracellular matrix formation can increase myocardial stiffness, causing ventricular systolic and diastolic dysfunction and HF [4]. Certain biomolecules mediating inflammatory and pathophysiological processes, such as myocardial fibrosis and remodeling, can be HF biomarkers [5, 6]. Fibroblasts, myofibroblasts, and extracellular matrix components actively shape and respond to atrial fibrosis. FGF2 is important in atrial fibrosis and is located in the cytoplasm, nucleus, and extracellular matrix in developmental and adult atria [17]. FGF2 is crucial for in vitro myocardial remodeling [18], and the FGF2-FGF receptor-1 axis is a potential therapeutic target for treating cardiac hypertrophy [19].

Similarly, FGF2 expression is reportedly upregulated in patients with pressure or volume overload, causing left or right ventricular hypertrophy [20], and increased FGF2 levels are closely associated with human atrial fibrosis [19]. Although the findings of previous studies [21, 22] suggested that FGF2 plays a pivotal role in atrial fibrosis and remodeling, the role of hi-FGF2 in mediating atrial extracellular matrix regulation, fibrosis, and remodeling is controversial. Li et al. [23] demonstrated that increased levels of hi-FGF2 were closely interrelated with fibrotic human atria and might accelerate atrial fibrosis via the mitogen-activated protein kinase signaling pathway. Conversely, in a mouse model of MI, the lack of low molecular weight FGF2 resulted in greater MI scar formation [19]. As opposed to the low molecular weight FGF2 subtype, hi-FGF2 induces cardiomyocyte hypertrophy in vitro [18]. Ling-Yue et al. [16] confirmed that hi-FGF2 concentration can help predict the occurrence of HF in patients with atrial fibrillation and is an independent risk factor. Here, we demonstrated that the circulating levels of FGF2 are specifically elevated in patients with HF. Although hi-FGF2 accounts for 70% of the total cardiac FGF2 [23], whether hi-FGF2 dominates the circulating level remains unknown. However, building on the results of this study, we speculate that the circulating FGF2 primarily exists as hi-FGF2. Further studies are needed to confirm this hypothesis.

The symptoms and signs of some non-vascular diseases, such as anemia, lung disease, kidney disease, and thyroid disease, may be clinically similar to those of HF. To explore FGF2 specificity, we enrolled healthy participants and patients having dyspnea without HF as controls. The results showed that patients with HF had significantly higher FGF2 levels than those having non-HF dyspnea and healthy controls. The AUC was 0.8963, demonstrating that FGF2 can distinguish HF from other causes of dyspnea and is specific for HF. The results were less affected by cardiac cell injury due to the exclusion of patients with recent acute MI. Therefore, FGF2 may be a specific, novel HF biomarker that facilitates more accurate HF diagnosis, detection, and monitoring of cardiac injury before it develops to an irreversible stage.

Our data revealed a negative correlation between FGF2 concentration and LVEF, indicating a possible relationship between FGF2 level and impaired cardiac function and that FGF2 level may stratify the risk for HF. The risk of HF increases as the NT-proBNP level increases [24], and the NT-proBNP level is closely related to HF severity, NYHA grade, end-diastolic pressure, and the degree of hemodynamic disturbance [25]. NT-proBNP level is a robust biomarker for predicting readmission and mortality in patients with acute decompensated HF [26]. It has great value for diagnosis and short- and long-term prognostic evaluation in patients with concurrent dyspnea and suspected or confirmed acute HF [27, 28]. In this study, we found that FGF2 level was moderately correlated with NT-proBNP level, indicating that FGF2 level may be associated with the prognosis of patients with HF and could potentially be used for their risk stratification. However, follow-up data are needed to support this conjecture.

This study had certain limitations. Although we included patients with non-HF dyspnea, the overall number of cases with missing data was small. Hence, larger studies are needed to increase the validity and reliability of the results. Our results suggest an association between FGF2 level and known, vital, prognostic HF parameters. However, using FGF2 levels for risk stratification in patients with HF remains to be validated with long-term follow-up assessment.

## Conclusions

This study found that FGF2 level may be a potential biomarker for distinguishing patients with HF from those with non-HF dyspnea. Additionally, increased FGF2 expression levels were correlated with vital prognostic parameters, such as increased NT-proBNP level and decreased LVEF.

### Supplementary Information

**Additional file 1: Table S1** ROC curve: HF vs non HF. **Table S2** Circulating levels of FGF2 in healthy controls (n=36) and HF cases with ejection fraction (EF)<45% (n=16) and EF>45% (n=64). **Table S3** Expression levels of FGF2 according to New York Heart Association cardiac function classification. **Table S4** Expression levels of FGF2 in different HF types. **Table S5** Expression levels of FGF2 in groups with different HF etiologies. **Table S6** Spearman correlation of FGF2 level with N-terminal pro-B-type natriuretic peptide level. **Table S7** Spearman correlation of FGF2 level with EF. **Figure S1** FGF2 levels in patients with HF(-) AF(-),HF(+)AF(-)and HF(+)AF(+).

## Data Availability

The datasets used and analyzed during the current study are available from the corresponding author on reasonable request.

## Abbreviations

AUC: Area under the receiver operating characteristic curve
CI: Confidence interval
FGF2: Fibroblast growth factor 2
HF: Heart failure
AF: Atrial fibrillation
hi-FGF2: High-molecular-weight fibroblast growth factor 2 isoform
LVEF: Left ventricular ejection fraction
MI: Myocardial infarction
NT-proBNT: N-terminal pro-B-type natriuretic peptide
NYHA: New York heart association
