# Supplementary material for: Serum fibroblast growth factor-2 levels complement vital biomarkers for diagnosing heart failure
Source: BMC Cardiovasc Disord. 2024 Feb 15;24:109. doi: 10.1186/s12872-024-03768-4 (PMC10868019; doi:10.1186/s12872-024-03768-4)

**Additional file**

Table S1.ROC curve: HF vs non HF

| HF | non HF | HF | non-HF dyspnea | Healthy controls |
| --- | --- | --- | --- | --- |
| 523 | 6.2 | 523 | 6.2 | 20.6 |
| 437.8 | 12.6 | 437.8 | 12.6 | 32 |
| 415.8 | 12.8 | 415.8 | 12.8 | 35.2 |
| 379.6 | 15.2 | 379.6 | 15.2 | 38.2 |
| 373.8 | 17.2 | 373.8 | 17.2 | 41.4 |
| 306.4 | 21.8 | 306.4 | 21.8 | 43.8 |
| 298.6 | 23 | 298.6 | 23 | 46.2 |
| 287.2 | 24.8 | 287.2 | 24.8 | 51 |
| 283.4 | 30.2 | 283.4 | 30.2 | 60 |
| 282.6 | 35.2 | 282.6 | 35.2 | 63.2 |
| 280.6 | 38 | 280.6 | 38 | 63.2 |
| 274 | 38.2 | 274 | 38.2 | 63.2 |
| 270.6 | 39.8 | 270.6 | 39.8 | 65.4 |
| 231.2 | 41.8 | 231.2 | 41.8 | 66 |
| 227 | 42.8 | 227 | 42.8 | 67.8 |
| 197.8 | 59.8 | 197.8 | 59.8 | 73 |
| 193 | 65 | 193 | 65 | 78.4 |
| 190.4 | 65.6 | 190.4 | 65.6 | 78.6 |
| 187.8 | 67 | 187.8 | 67 | 79.2 |
| 183.8 | 67.8 | 183.8 | 67.8 | 79.2 |
| 182.2 | 70.6 | 182.2 | 70.6 | 79.6 |
| 178.8 | 72.8 | 178.8 | 72.8 | 80.6 |
| 175.2 | 74 | 175.2 | 74 | 81 |
| 169.6 | 75.8 | 169.6 | 75.8 | 82 |
| 169 | 76.8 | 169 | 76.8 | 83.2 |
| 168.8 | 77.4 | 168.8 | 77.4 | 83.6 |
| 166.6 | 83.6 | 166.6 | 83.6 | 86.6 |
| 163 | 87.8 | 163 | 87.8 | 87.4 |
| 158.4 | 92.8 | 158.4 | 92.8 | 89 |
| 154.2 | 95.6 | 154.2 | 95.6 | 97.6 |
| 154 | 97.6 | 154 | 97.6 | 101 |
| 145.6 | 97.8 | 145.6 | 97.8 | 102.6 |
| 142.4 | 98.8 | 142.4 | 98.8 | 103.4 |
| 141.8 | 100.6 | 141.8 | 100.6 | 105.8 |
| 141.4 | 20.6 | 141.4 |  | 113 |
| 132 | 32 | 132 |  | 117.2 |
| 130 | 35.2 | 130 |  |  |
| 129.8 | 38.2 | 129.8 |  |  |
| 129.4 | 41.4 | 129.4 |  |  |
| 126.8 | 43.8 | 126.8 |  |  |
| 124.4 | 46.2 | 124.4 |  |  |
| 121 | 51 | 121 |  |  |
| 120.6 | 60 | 120.6 |  |  |
| 118 | 63.2 | 118 |  |  |
| 115.6 | 63.2 | 115.6 |  |  |
| 115 | 63.2 | 115 |  |  |
| 114.6 | 65.4 | 114.6 |  |  |

**Table S2.** Circulating levels of FGF2 in healthy controls (n=36) and HF cases with ejection fraction (EF)<45% (n=16) and EF>45% (n=64)

| Healthy | HF with EF<45% | HF with EF>45% |
| --- | --- | --- |
| 20.6 | 373.8 | 523 |
| 32 | 282.6 | 437.8 |
| 35.2 | 274 | 415.8 |
| 38.2 | 270.6 | 379.6 |
| 41.4 | 187.8 | 306.4 |
| 43.8 | 154.2 | 298.6 |
| 46.2 | 154 | 287.2 |
| 51 | 142.4 | 283.4 |
| 60 | 130 | 280.6 |
| 63.2 | 106.8 | 231.2 |
| 63.2 | 97.4 | 227 |
| 63.2 | 96.6 | 197.8 |
| 65.4 | 83 | 193 |
| 66 | 78.4 | 190.4 |
| 67.8 | 78 | 183.8 |
| 73 | 71.6 | 182.2 |
| 78.4 |  | 178.8 |
| 78.6 |  | 175.2 |
| 79.2 |  | 169.6 |
| 79.2 |  | 169 |
| 79.6 |  | 168.8 |
| 80.6 |  | 166.6 |
| 81 |  | 163 |
| 82 |  | 158.4 |
| 83.2 |  | 145.6 |
| 83.6 |  | 141.8 |
| 86.6 |  | 141.4 |
| 87.4 |  | 132 |
| 89 |  | 129.8 |
| 97.6 |  | 129.4 |
| 101 |  | 126.8 |
| 102.6 |  | 124.4 |
| 103.4 |  | 121 |
| 105.8 |  | 120.6 |
| 113 |  | 118 |
| 117.2 |  | 115.6 |
|  |  | 115 |
|  |  | 114.6 |
|  |  | 114.4 |
|  |  | 110.4 |
|  |  | 108 |
|  |  | 107.4 |
|  |  | 103.2 |
|  |  | 103 |
|  |  | 100.2 |
|  |  | 96 |
|  |  | 94.2 |
|  |  | 93 |
|  |  | 87.6 |
|  |  | 87.2 |
|  |  | 85.8 |
|  |  | 83.8 |
|  |  | 83.4 |
|  |  | 82.8 |
|  |  | 82 |
|  |  | 81.6 |
|  |  | 80.6 |
|  |  | 77.4 |
|  |  | 75.8 |
|  |  | 75.2 |
|  |  | 73.8 |
|  |  | 71.4 |
|  |  | 71.4 |
|  |  | 71 |

**Table S3.** Expression levels of FGF2 according to New York Heart Association cardiac function classification.

| Healthy | NYHA 1 | NYHA 2 | NYHA 3 | NYHA 4 |
| --- | --- | --- | --- | --- |
| 20.6 | 283.4 | 437.8 | 415.8 | 187.8 |
| 32 | 197.8 | 298.6 | 379.6 | 168.8 |
| 35.2 | 183.8 | 280.6 | 373.8 | 158.4 |
| 38.2 | 154.2 | 190.4 | 282.6 | 141.8 |
| 41.4 | 129.4 | 175.2 | 270.6 | 107.4 |
| 43.8 | 121 | 169 | 231.2 | 78.4 |
| 46.2 | 114.4 | 166.6 | 227 | 71.6 |
| 51 | 83.8 | 132 | 182.2 | 71 |
| 60 | 83.4 | 115.6 | 145.6 |  |
| 63.2 | 80.6 | 115 | 142.4 |  |
| 63.2 | 71.4 | 106.8 | 129.8 |  |
| 63.2 |  | 103.2 | 120.6 |  |
| 65.4 |  | 103 | 118 |  |
| 66 |  | 100.2 | 110.4 |  |
| 67.8 |  | 96 | 108 |  |
| 73 |  | 87.6 | 97.4 |  |
| 78.4 |  | 87.2 | 94.2 |  |
| 78.6 |  | 85.8 | 93 |  |
| 79.2 |  | 82 | 83 |  |
| 79.2 |  | 78 | 81.6 |  |
| 79.6 |  | 75.8 | 77.4 |  |
| 80.6 |  | 75.2 |  |  |
| 81 |  |  |  |  |
| 82 |  |  |  |  |
| 83.2 |  |  |  |  |
| 83.6 |  |  |  |  |
| 86.6 |  |  |  |  |
| 87.4 |  |  |  |  |
| 89 |  |  |  |  |
| 97.6 |  |  |  |  |
| 101 |  |  |  |  |
| 102.6 |  |  |  |  |
| 103.4 |  |  |  |  |
| 105.8 |  |  |  |  |
| 113 |  |  |  |  |
| 117.2 |  |  |  |  |

**Table S4.** Expression levels of FGF2 in different HF types.

| Healthy | HFrEF | HFmrEF | HFpEF |
| --- | --- | --- | --- |
| 20.6 | 373.8 | 187.8 | 523 |
| 32 | 282.6 | 169.6 | 437.8 |
| 35.2 | 274 | 154.2 | 415.8 |
| 38.2 | 270.6 | 120.6 | 379.6 |
| 41.4 | 154 | 115 | 306.4 |
| 43.8 | 142.4 | 108 | 298.6 |
| 46.2 | 130 | 81.6 | 287.2 |
| 51 | 106.8 | 71.4 | 283.4 |
| 60 | 97.4 |  | 280.6 |
| 63.2 | 96.6 |  | 231.2 |
| 63.2 | 83 |  | 227 |
| 63.2 | 78.4 |  | 197.8 |
| 65.4 | 78 |  | 193 |
| 66 | 71.6 |  | 190.4 |
| 67.8 |  |  | 183.8 |
| 73 |  |  | 182.2 |
| 78.4 |  |  | 178.8 |
| 78.6 |  |  | 175.2 |
| 79.2 |  |  | 169 |
| 79.2 |  |  | 168.8 |
| 79.6 |  |  | 166.6 |
| 80.6 |  |  | 163 |
| 81 |  |  | 158.4 |
| 82 |  |  | 145.6 |
| 83.2 |  |  | 141.8 |
| 83.6 |  |  | 141.4 |
| 86.6 |  |  | 132 |
| 87.4 |  |  | 129.8 |
| 89 |  |  | 129.4 |
| 97.6 |  |  | 126.8 |
| 101 |  |  | 124.4 |
| 102.6 |  |  | 121 |
| 103.4 |  |  | 118 |
| 105.8 |  |  | 115.6 |
| 113 |  |  | 114.6 |
| 117.2 |  |  | 114.4 |
|  |  |  | 110.4 |
|  |  |  | 107.4 |
|  |  |  | 103.2 |
|  |  |  | 103 |
|  |  |  | 100.2 |
|  |  |  | 96 |
|  |  |  | 94.2 |
|  |  |  | 93 |
|  |  |  | 87.6 |
|  |  |  | 87.2 |
|  |  |  | 85.8 |
|  |  |  | 83.8 |
|  |  |  | 83.4 |
|  |  |  | 82.8 |
|  |  |  | 82 |
|  |  |  | 80.6 |
|  |  |  | 77.4 |
|  |  |  | 75.8 |
|  |  |  | 75.2 |
|  |  |  | 73.8 |
|  |  |  | 71.4 |
|  |  |  | 71 |

**Table S5.** Expression levels of FGF2 in groups with different HF etiologies.

| Healthy | Ischemic HF | Non-ischemic HF |
| --- | --- | --- |
| 20.6 | 523 | 415.8 |
| 32 | 437.8 | 379.6 |
| 35.2 | 306.4 | 373.8 |
| 38.2 | 283.4 | 298.6 |
| 41.4 | 282.6 | 287.2 |
| 43.8 | 280.6 | 274 |
| 46.2 | 227 | 270.6 |
| 51 | 197.8 | 231.2 |
| 60 | 190.4 | 193 |
| 63.2 | 183.8 | 187.8 |
| 63.2 | 182.2 | 178.8 |
| 63.2 | 175.2 | 169.6 |
| 65.4 | 169 | 168.8 |
| 66 | 166.6 | 163 |
| 67.8 | 158.4 | 145.6 |
| 73 | 154.2 | 141.8 |
| 78.4 | 154 | 126.8 |
| 78.6 | 142.4 | 124.4 |
| 79.2 | 141.4 | 120.6 |
| 79.2 | 132 | 118 |
| 79.6 | 130 | 114.6 |
| 80.6 | 129.8 | 110.4 |
| 81 | 129.4 | 108 |
| 82 | 121 | 107.4 |
| 83.2 | 115.6 | 106.8 |
| 83.6 | 115 | 97.4 |
| 86.6 | 114.4 | 94.2 |
| 87.4 | 103.2 | 85.8 |
| 89 | 103 | 82.8 |
| 97.6 | 100.2 | 81.6 |
| 101 | 96.6 | 77.4 |
| 102.6 | 96 | 75.8 |
| 103.4 | 93 | 75.2 |
| 105.8 | 87.6 | 73.8 |
| 113 | 87.2 | 71.4 |
| 117.2 | 83.8 | 71.4 |
|  | 83.4 |  |
|  | 83 |  |
|  | 82 |  |
|  | 80.6 |  |
|  | 78.4 |  |
|  | 78 |  |
|  | 71.6 |  |
|  | 71 |  |

**Table S6.** Spearman correlation of FGF2 level with N-terminal pro-B-type natriuretic peptide level

| FGF2 | NTproBNP |
| --- | --- |
| 523 |  |
| 437.8 | 451 |
| 415.8 | 1052 |
| 379.6 | 8155 |
| 373.8 | 5101 |
| 306.4 | 4289 |
| 298.6 | 379 |
| 287.2 |  |
| 283.4 | 787 |
| 282.6 | 10000 |
| 280.6 | 381 |
| 274 | 5814 |
| 270.6 | 10000 |
| 231.2 | 3310 |
| 227 | 100 |
| 197.8 | 2789 |
| 193 | 7998 |
| 190.4 |  |
| 187.8 | 3596 |
| 183.8 | 100 |
| 182.2 | 10000 |
| 178.8 | 1325 |
| 175.2 | 800 |
| 169.6 | 654 |
| 169 |  |
| 168.8 | 9705 |
| 166.6 |  |
| 163 | 2359 |
| 158.4 | 10000 |
| 154.2 | 4928 |
| 154 | 10000 |
| 145.6 | 538 |
| 142.4 | 8213 |
| 141.8 | 704 |
| 141.4 | 4934 |
| 132 |  |
| 130 | 10000 |
| 129.8 |  |
| 129.4 |  |
| 126.8 | 100 |
| 124.4 |  |
| 121 |  |
| 120.6 | 10000 |
| 118 |  |
| 115.6 | 100 |
| 115 | 2414 |
| 114.6 | 684 |
| 114.4 |  |
| 110.4 | 10000 |
| 108 |  |
| 107.4 | 403 |
| 106.8 | 5735 |
| 103.2 |  |
| 103 |  |
| 100.2 | 319 |
| 97.4 | 10000 |
| 96.6 |  |
| 96 |  |
| 94.2 |  |
| 93 |  |
| 87.6 |  |
| 87.2 |  |
| 85.8 |  |
| 83.8 |  |
| 83.4 | 1183 |
| 83 | 1511 |
| 82.8 | 9683 |
| 82 |  |
| 81.6 | 8536 |
| 80.6 | 295 |
| 78.4 | 855 |
| 78 |  |
| 77.4 | 7392 |
| 75.8 |  |
| 75.2 |  |
| 73.8 |  |
| 71.6 | 3610 |
| 71.4 |  |
| 71.4 |  |
| 71 | 5198 |
| 6.2 |  |
| 12.6 | 563 |
| 12.8 |  |
| 15.2 |  |
| 17.2 | 100 |
| 21.8 |  |
| 23 |  |
| 24.8 |  |
| 30.2 | 261 |
| 35.2 |  |
| 38 |  |
| 38.2 |  |
| 39.8 | 128 |
| 41.8 | 100 |
| 42.8 |  |
| 59.8 | 100 |
| 65 | 204 |
| 65.6 | 100 |
| 67 | 100 |
| 67.8 |  |
| 70.6 |  |
| 72.8 | 100 |
| 74 |  |
| 75.8 | 100 |
| 76.8 |  |
| 77.4 | 100 |
| 83.6 | 284 |
| 87.8 | 100 |
| 92.8 | 100 |
| 95.6 |  |
| 97.6 | 262 |
| 97.8 |  |
| 98.8 | 122 |
| 100.6 | 100 |
| 20.6 | 100 |
| 32 | 100 |
| 35.2 | 100 |
| 38.2 | 100 |
| 41.4 | 100 |
| 43.8 | 100 |
| 46.2 | 100 |
| 51 | 100 |
| 60 | 100 |
| 63.2 | 100 |
| 63.2 | 100 |
| 63.2 | 100 |
| 65.4 | 100 |
| 66 | 100 |
| 67.8 | 100 |
| 73 | 100 |
| 78.4 | 100 |
| 78.6 | 100 |
| 79.2 | 100 |
| 79.2 | 100 |
| 79.6 | 100 |
| 80.6 | 100 |
| 81 | 100 |
| 82 | 100 |
| 83.2 | 100 |
| 83.6 | 100 |
| 86.6 | 100 |
| 87.4 | 100 |
| 89 | 100 |
| 97.6 | 100 |
| 101 | 100 |
| 102.6 | 100 |
| 103.4 | 100 |
| 105.8 | 100 |
| 113 | 100 |
| 117.2 | 100 |

**Table S7.** Spearman correlation of FGF2 level with EF .

| EF | Hi-FGF2 |
| --- | --- |
| 51 | 523 |
| 61 | 437.8 |
| 62 | 415.8 |
| 56 | 379.6 |
| 27 | 373.8 |
| 59 | 306.4 |
| 60 | 298.6 |
| 60 | 287.2 |
| 58 | 283.4 |
| 24 | 282.6 |
| 56 | 280.6 |
| 40 | 274 |
| 15 | 270.6 |
| 57 | 231.2 |
| 57 | 227 |
| 62 | 197.8 |
| 50 | 193 |
| 57 | 190.4 |
| 42 | 187.8 |
| 57 | 183.8 |
| 51 | 182.2 |
| 55 | 178.8 |
| 51 | 175.2 |
| 48 | 169.6 |
| 69 | 169 |
| 58 | 168.8 |
| 61 | 166.6 |
| 52 | 163 |
| 56 | 158.4 |
| 41 | 154.2 |
| 26 | 154 |
| 66 | 145.6 |
| 30 | 142.4 |
| 54 | 141.8 |
| 63 | 141.4 |
| 54 | 132 |
| 30 | 130 |
| 61 | 129.8 |
| 64 | 129.4 |
| 65 | 126.8 |
| 69 | 124.4 |
| 65 | 121 |
| 46 | 120.6 |
| 64 | 118 |
| 64 | 115.6 |
| 47 | 115 |
| 56 | 114.6 |
| 54 | 114.4 |
| 61 | 110.4 |
| 49 | 108 |
| 67 | 107.4 |
| 24 | 106.8 |
| 51 | 103.2 |
| 67 | 103 |
| 67 | 100.2 |
| 30 | 97.4 |
| 32 | 96.6 |
| 65 | 96 |
| 60 | 94.2 |
| 62 | 93 |
| 52 | 87.6 |
| 65 | 87.2 |
| 61 | 85.8 |
| 66 | 83.8 |
| 64 | 83.4 |
| 39 | 83 |
| 51 | 82.8 |
| 62 | 82 |
| 46 | 81.6 |
| 56 | 80.6 |
| 40 | 78.4 |
| 40 | 78 |
| 61 | 77.4 |
| 55 | 75.8 |
| 61 | 75.2 |
| 63 | 73.8 |
| 26 | 71.6 |
| 48 | 71.4 |
| 62 | 71.4 |
| 55 | 71 |
| 63 | 6.2 |
|  | 12.6 |
| 67 | 12.8 |
| 58 | 15.2 |
| 66 | 17.2 |
| 66 | 21.8 |
| 63 | 23 |
| 68 | 24.8 |
| 69 | 30.2 |
| 59 | 35.2 |
| 61 | 38 |
| 66 | 38.2 |
| 62 | 39.8 |
| 66 | 41.8 |
| 62 | 42.8 |
| 65 | 59.8 |
| 53 | 65 |
| 67 | 65.6 |
| 68 | 67 |
|  | 67.8 |
| 62 | 70.6 |
| 63 | 72.8 |
| 64 | 74 |
| 62 | 75.8 |
| 68 | 76.8 |
| 57 | 77.4 |
| 71 | 83.6 |
| 69 | 87.8 |
| 63 | 92.8 |
| 68 | 97.6 |
| 48 | 97.8 |
| 57 | 98.8 |
| 64 | 100.6 |
| 67 | 20.6 |
| 72 | 32 |
| 70 | 41.4 |
| 68 | 43.8 |
| 68 | 65.4 |
| 65 | 79.2 |

**Figure legend**

Figure S1. FGF2 levels in patients with HF(-) AF(-),HF(+)AF(-)and HF(+)AF(+)

*ns p*＞0.05; **** p* <0.001. Abbreviations: FGF2, fibroblast growth factor 2; HF, heart failure; AF, atrial fibrillation


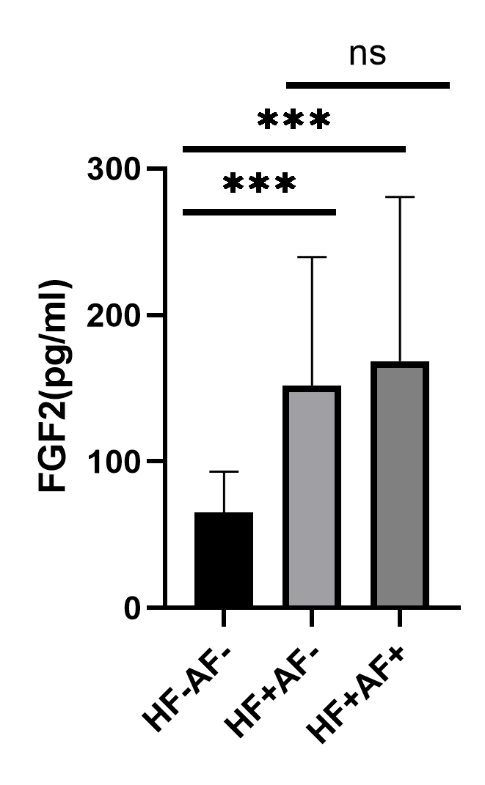

Supplement: Supplementary file 1 — Additional file 1: Table S1 ROC curve: HF vs non HF. Table S2 Circulating levels of FGF2 in healthy controls (n=36) and HF cases with ejection fraction (EF)<45% (n=16) and EF>45% (n=64). Table S3 Expression levels of FGF2 according to New York Heart Association cardiac function classification. Table S4 Expression levels of FGF2 in different HF types. Table S5 Expression levels of FGF2 in groups with different HF etiologies. Table S6 Spearman correlation of FGF2 level with N-terminal pro-B-type natriuretic peptide level. Table S7 Spearman correlation of FGF2 level with EF. Figure S1 FGF2 levels in patients with HF(-) AF(-),HF(+)AF(-)and HF(+)AF(+). [file 12872_2024_3768_MOESM1_ESM.docx]
